# Precious-metal free photoelectrochemical water splitting with immobilised molecular Ni and Fe redox catalysts†

**DOI:** 10.1039/c5sc04863j

**Published:** 2016-02-12

**Authors:** Timothy E. Rosser, Manuela A. Gross, Yi-Hsuan Lai, Erwin Reisner

**Affiliations:** a Christian Doppler Laboratory for Sustainable SynGas Chemistry, Department of Chemistry, University of Cambridge Lensfield Road Cambridge CB1 2EW UK reisner@ch.cam.ac.uk

## Abstract

Splitting water into hydrogen and oxygen with molecular catalysts and light has been a long-established challenge. Approaches in homogeneous systems have been met with little success and the integration of molecular catalysts in photoelectrochemical cells is challenging due to inaccessibility and incompatibility of functional hybrid molecule/material electrodes with long-term stability in aqueous solution. Here, we present the first example of light-driven water splitting achieved with precious-metal-free molecular catalysts driving both oxygen and hydrogen evolution reactions. Mesoporous TiO_2_ was employed as a low-cost scaffold with long-term stability for anchoring a phosphonic acid-modified nickel(ii) bis-diphosphine catalyst (NiP) for electrocatalytic proton reduction. A turnover number of 600 mol H_2_ per mol NiP was achieved after 8 h controlled-potential electrolysis at a modest overpotential of 250 mV. X-ray photoelectron, UV-vis and IR spectroscopies confirmed that the molecular structure of the Ni catalyst remains intact after prolonged hydrogen production, thereby reasserting the suitability of molecular catalysts in the development of effective, hydrogen-evolving materials. The relatively mild operating conditions of a pH 3 aqueous solution allowed this molecule-catalysed cathode to be combined with a molecular Fe(ii) catalyst-modified WO_3_ photoanode in a photoelectrochemical cell. Water splitting into H_2_ and O_2_ was achieved under solar light illumination with an applied bias of >0.6 V, which is below the thermodynamic potential (1.23 V) for water splitting and therefore allowed the storage of solar energy in the fuel H_2_.

## Abstract 2

Splitting water into hydrogen and oxygen with 3d transition metal molecular catalysts and light has been accomplished.

## Graphical abstract

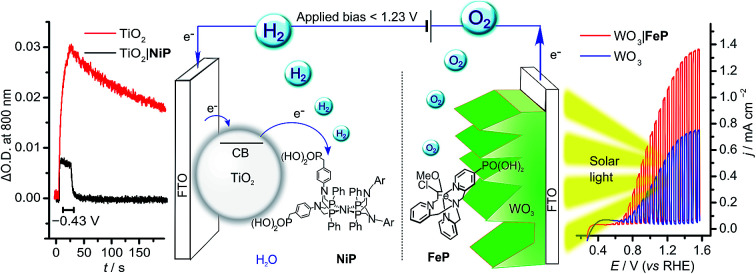

## Introduction

Splitting water into hydrogen and oxygen using insolation, a process considered as ‘artificial photosynthesis’, is viewed as a promising sustainable solution to meeting the increasing global demand for transportable fuel and storable renewable energy. The reliance on the long excited state lifetimes^1^ and excellent catalytic properties^2,3^ characteristic of components based on low-abundance precious metal elements such as Ru and Pt remains a barrier to low-cost water splitting.^4,5^ Consequently, only a handful of systems that achieve full water splitting make use of catalysts made from only Earth-abundant elements.^6–8^ Of these, none rely on synthetic molecular catalysts driving both the H_2_ and O_2_ evolution half reactions, and as such the realisation of this goal remains a significant challenge.^9,10^

The specific interest in molecular catalysts arises from the precise control afforded by modern synthetic chemistry over the individual catalytic centres, and therefore the opportunity to study the chemical and structural influences on catalysis.^11–13^ Indeed, there have been cases where only molecular catalysts, and not noble metal nanoparticles, are found to perform H_2_ evolution.^14^ Thus far, H_2_ evolution with molecular catalysts driving the reduction and oxidation reactions in homogeneous solution has only been achieved in the context of oxidation of an organic substrate.^15,16^ Thus, homogeneous approaches have failed to date in demonstrating full splitting of H_2_O into H_2_ and O_2_.

A photoelectrochemical (PEC) approach to water splitting utilising immobilised molecular catalysts has many advantages. It allows efficient use of highly active and selective catalytic centres (‘single-site-catalysis’), can overcome kinetic restrictions from diffusion limitations, separates the redox half-reactions to avoid quenching mechanisms and allows separation of the gaseous products, as well as providing a platform for the systematic study of molecular catalysts under aqueous conditions without the requirement of water-solubility.^11,17^ Recently, the first molecule-catalysed tandem PEC cells with an immobilised Co-based catalyst driving a dye-sensitised NiO photocathode and a Ru catalyst on a dye-sensitised photoanode have demonstrated full water splitting.^18,19^ However, this PEC cell relies on a precious metal (Ru) water oxidation catalyst and the lability of the axial pyridine on the cobalt H_2_ evolution catalyst limits the long-term applicability of the H_2_-evolving photocathode.

**Scheme 1:**
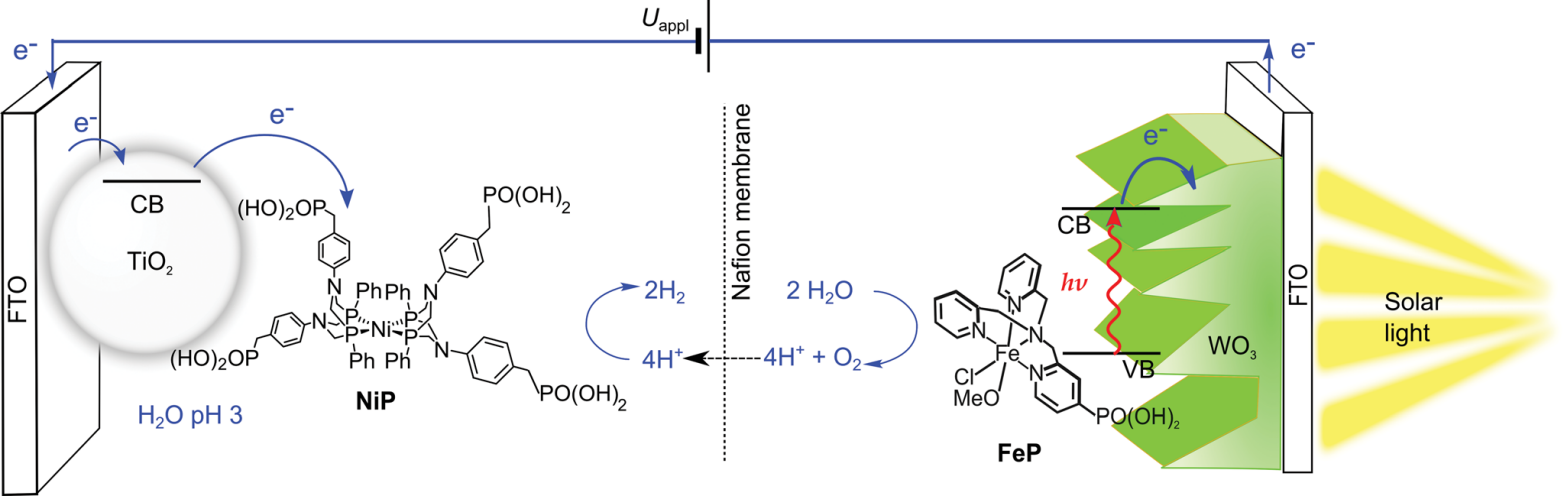
Schematic representation of PEC water splitting with the TiO_2_|NiP hydrogen evolution cathode wired to the WO_3_|FeP oxygen evolution photoanode in an aqueous electrolyte solution at pH 3.

In this study, we present the first example of molecular catalyst-enabled water splitting using only Earth-abundant elements (Scheme 1). We have selected the Ni(ii) bis-diphosphine class of H_2_ evolution catalyst due to its high activity in both aqueous^20,21^ and non-aqueous^22^ conditions and lack of a labile ligand that could undergo hydrolysis or displacement during catalysis, and immobilised a phosphonate-bearing example on a mesoporous TiO_2_ electrode. We have studied the activity and stability of this hybrid cathode, finding both to be excellent in mild aqueous conditions, which are essential for combination with a photoanode for solar water splitting. An Fe(ii)-based molecular catalyst immobilised on WO_3_ has shown a well-characterised increase in activity and selectivity for O_2_ evolution in mildly acidic aqueous conditions compared to the unmodified electrodes,^23^ and as such was have combined the TiO_2_ hybrid cathode with a Fe-catalyst modified WO_3_ photoanode in a PEC water splitting cell.

## Results and discussion

### Hybrid H_2_ evolution cathode

Ni(ii) bis(diphosphine) complexes are a family of bio-inspired proton reduction catalysts,^22,24,25^ and the phosphonate-bearing catalyst NiP (Scheme 1) ranks among the most active precious-metal free molecular H_2_ evolution catalysts in aqueous conditions.^14,20,26^NiP was reported with turnover numbers (TONs) in excess of 700 mol H_2_ per mol NiP in photocatalytic schemes using a Ru(ii)-based dye and ascorbic acid as an electron donor, and has been demonstrated as a homogeneous electrocatalyst in pH 4.5 aqueous solution.^27^NiP features phosphonic acid groups, which are well-established for effective binding to TiO_2_ under mildly acidic aqueous conditions,^28–30^ through a variety of modes such as P–O–Ti and hydrogen bonding, depending on the exposed TiO_2_ facet.^31^ These anchoring groups and the low ligand lability of NiP make it an ideal candidate for immobilisation on metal oxide electrodes for single-site heterogeneous proton reduction.

Attachment of molecular H_2_-evolving catalysts bearing phosphonic acid groups to metal oxides in acidic and pH neutral conditions has been demonstrated,^20,32^ including the electrochemical reduction of aqueous protons with cobalt(iii) catalysts immobilised onto mesoporous indium-doped tin oxide (ITO) electrodes.^11,33^ The performance of these electrodes, however, was low due to instability of ITO under reducing conditions, as well as the intrinsically low stability of the cobalt catalyst during turnover. NiP displays substantially higher activity than the previously employed Co-based catalysts and we replaced the electrodegrading ITO cathode with robust TiO_2_. Although TiO_2_ is often considered as a classical insulator and therefore unsuitable as electrode material, it has previously been used for electrocatalytic proton reduction with immobilised H_2_-cycling enzymes known as hydrogenases,^34,35^ and we aim here to establish its use as an electrode substrate for synthetic molecular catalysts such as NiP for long-term reductive electrocatalysis.

NiP was synthesised and characterised as described previously,^20^ and mesoporous TiO_2_ (mesoTiO_2_) electrodes were prepared by doctor blading a suspension of P25 TiO_2_ (8 : 2 anatase : rutile ratio, 25 nm average particle size) onto an FTO-coated glass substrates, followed by annealing at 450 °C. MesoTiO_2_ electrodes with a geometrical surface area of 1.0 cm^2^ were employed and scanning electron microscopy (SEM) revealed a film thickness of 4 μm (Fig. S1†). Modification with NiP was achieved by submersion of mesoTiO_2_ in a methanol solution of the Ni(ii) compound (0.5 mM) for 18 h at room temperature, followed by rinsing with methanol and drying under a stream of N_2_. The amount of NiP per geometric surface area of the TiO_2_ electrode was determined as 14.6 ± 2.0 nmol cm^−2^ by spectrophotometry (at *λ*_abs_ = 257 and 300 nm) following desorption of the catalyst from TiO_2_ with aqueous NaOH (0.1 M). The loading of NiP is in agreement with previous results, where a surface coverage of 53 nmol cm^−2^ was observed for a phosphonic acid-modified ruthenium complex on 10 μm thick mesoporous TiO_2_ electrodes.^36^

**Fig. 1:**
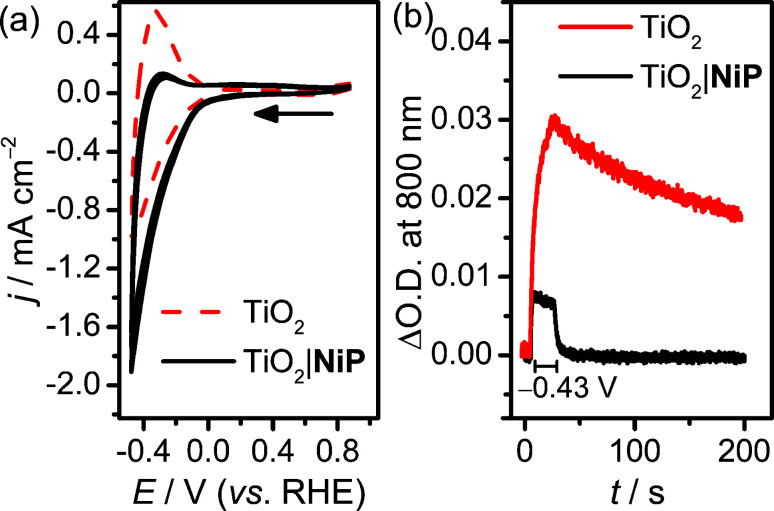
(a) CVs of NiP immobilised on mesoTiO_2_ (solid black line, TiO_2_|NiP) and unmodified mesoTiO_2_ (dashed red line) performed at *ν* = 100 mV s^−1^ (the arrow indicates the start of the experiment). (b) Spectroelectrochemistry of the same electrodes monitoring the absorbance change (ΔO.D.) at *λ* = 800 nm before (no applied potential), during CPE at *E*_appl_ = −0.43 V *vs.* RHE for 20 s (starts at 6 s), and following CPE (no applied potential). Conditions: a Ag/AgCl reference electrode and a Pt mesh counter electrode in aqueous Na_2_SO_4_ (0.1 M) solution at pH 3, N_2_ atmosphere and room temperature.

Cyclic voltammograms (CVs) of the resultant NiP-modified mesoTiO_2_ (TiO_2_|NiP) electrodes and bare mesoTiO_2_ in aqueous pH 3 electrolyte solution (0.1 M Na_2_SO_4_) are shown in Fig. 1a, and suggest H_2_ evolution activity of the immobilised Ni(ii) catalyst. The CVs of the unmodified TiO_2_ electrode show a typical ‘trumpet plot’ response as expected for a semiconductor electrode.^37^ The reductive current observed upon the cathodic scan (*Q* = −0.6 mC at *ν* = 100 mV s^−1^), with an onset of approximately −0.1 V *vs.* the reversible hydrogen electrode (RHE), was followed by an oxidising current (*Q* = +0.4 mC) in the anodic scan. This observation was attributed to filling and emptying of the conduction band (CB) of TiO_2_. At pH 3, after modification with NiP, the oxidative current in the anodic scan is substantially diminished, with the charge ratio for the cathodic to anodic scans higher than 15 : 1, consistent with the consumption of the electrons from the CB of TiO_2_ for proton reduction catalysis.

Evidence that the electrons are transferred to the catalyst *via* the CB of TiO_2_ was obtained by spectroelectrochemistry.^38^ When an applied potential, *E*_appl_, of −0.43 V *vs.* RHE was applied to TiO_2_ electrodes, a blue colour was observed, and the corresponding increase in absorbance between 600 and 900 nm in the UV-vis spectrum is shown in Fig. S2a.† We assigned this absorption to d–d transitions in Ti^3+^, which is formed by filling the CB of TiO_2_.^39,40^ When treated with NiP, the increase in absorbance between 600 and 900 nm was still observed at *E*_appl_ = −0.43 V *vs.* RHE (Fig. S2b†), but to a lesser extent, due to a lower steady-state concentration of electrons in the CB. To study the release of CB electrons to NiP, the time-resolved absorbance^38^ of an unmodified and NiP-modified mesoTiO_2_ electrode was monitored at *λ* = 800 nm with *E*_appl_ = −0.43 V *vs.* RHE for 20 s, followed by a return to no applied potential (Fig. 1b). In the absence of NiP, the CB electrons are only slowly released from TiO_2_ after charging at −0.43 V *vs.* RHE, which is consistent with the oxidising (discharging) current observed in the return scan of the CV in Fig. 1a. In the presence of NiP on mesoTiO_2_, the absorbance at *λ* = 800 nm decayed to its original value within a few seconds (*τ*_1/2_ = 2.3 s) of the potential being removed, supporting the efficient release of CB electrons to NiP.

**Fig. 2:**
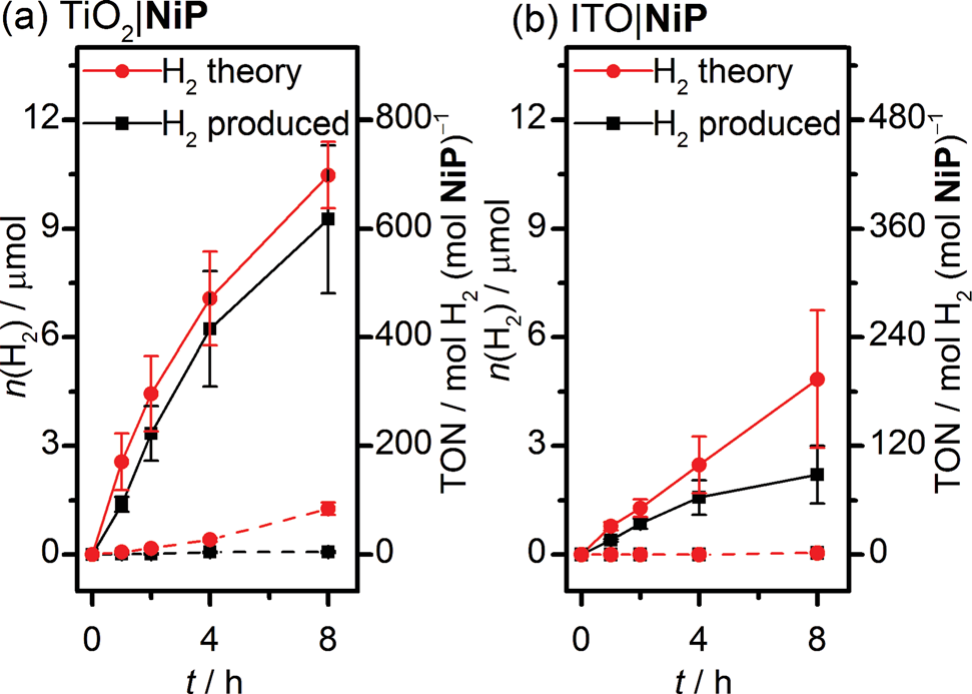
Amount of H_2_ generated over time during CPE of NiP-modified (solid line) and unmodified (dashed line) with (a) mesoTiO_2_ and (b) mesoITO electrodes at *E*_appl_ = −0.25 V *vs.* RHE. The theoretical amount of H_2_ was calculated based on 100% faradaic yield and the experimentally produced H_2_ was quantified by gas chromatography. Conditions: a Ag/AgCl reference electrode and a Pt mesh counter electrode in aqueous Na_2_SO_4_ (0.1 M) solution at pH 3, N_2_ atmosphere and room temperature.

Sustained electrocatalytic H_2_ production by the TiO_2_|NiP cathode was confirmed by controlled-potential electrolysis (CPE) in pH 3 aqueous electrolyte solution, and the amount of H_2_ produced alongside the theoretical maximum (assuming 100% faradaic efficiency) is shown in Fig. 2a. MesoTiO_2_ electrodes modified with NiP were held at *E*_appl_ = −0.25 V *vs.* RHE in a two-compartment electrolytic cell for 8 h, during which time a charge of 2.02 ± 0.17 C passed. The current decayed slowly during the first few hours, which is consistent with the good stability of the molecular catalyst in aqueous solution (Fig. S3a†).^20,27^ Analysis of the headspace of the electrochemical cell by gas chromatography revealed that 9.26 ± 2.1 μmol of H_2_ was produced after 8 h, which corresponds to a faradaic yield of 88 ± 17% and a Ni-based turnover number with a lower limit of 600 (assuming that all NiP remained electroactive on the electrode during CPE).

The fraction of adsorbed catalyst and molecular integrity were study by UV-vis absorption spectroscopy after electrolysis. NiP was desorbed from mesoTiO_2_ with NaOH (0.1 M) after 8 h CPE and the UV-vis spectrum of the resultant solution matched a reference spectrum recorded from freshly-modified electrodes treated in the same way, suggesting minimal degradation of the ligand framework of NiP (Fig. S4a†). The loading of NiP after CPE was 10.9 ± 2.5 nmol cm^−2^, which corresponds to 75% of that measured on the freshly-modified electrodes, thus demonstrating strong attachment between the phosphonate anchor and the TiO_2_ surface even under reducing conditions.

Further evidence for the retention of molecular NiP after CPE was obtained by attenuated total reflectance Fourier transform infrared (ATR-FTIR) spectroscopy. Fig. S4b† shows the FTIR spectra of NiP powder, TiO_2_|NiP before and after 4 h CPE at −0.25 V *vs.* RHE, and TiO_2_ (treated with Na_2_SO_4_ electrolyte solution). Stretches at 1260 cm^−1^ (P=O), 1440 and 1510 cm^−1^ (NiP ligand) were present in the reference NiP spectrum and TiO_2_|NiP before and after CPE, but not the TiO_2_ background, reasserting the molecular integrity of the catalyst surviving many turnovers on the electrode surface.

MesoTiO_2_ displayed substantially improved performance to mesoITO, which has previously been used as a substrate for a phosphonate-bearing Co-based catalyst.^33^ We prepared mesoporous ITO electrodes (for synthetic details see the Experimental section) with a NiP loading per geometric surface area of 25 nmol cm^−2^. Although the CV supports a catalytic current for ITO|NiP in an aqueous pH 3 electrolyte solution (Fig. S5†), CPE at *E*_appl_ = −0.25 V *vs.* RHE (Fig. S3b†) shows the formation of only 2.2 ± 0.8 μmol of H_2_ after 8 h with a faradaic efficiency of 49 ± 14% (Fig. 2b). The low faradaic efficiency is attributed to competing reductive degradation of the ITO, thus demonstrating the fragility of the ITO electrodes under reducing conditions compared with TiO_2_.

Electrocatalytic activity of TiO_2_|NiP was highest at pH 3 and a lower performance was observed in electrochemical experiments in pH 4 and pH 2 electrolyte solutions. The CV of TiO_2_|NiP at pH 4 is shown in Fig. S6a† and it does not display the same loss of oxidative discharging of the CB as observed at pH 3 (Fig. 1a). This implies loss of performance, which is corroborated by a lower H_2_ production rate during CPE at *E*_appl_ = −0.33 V *vs.* RHE (Fig. S6b†). When electrolysis was performed at pH 2 under otherwise the same conditions, activity was again observed to be lower than at pH 3 (Fig. S5b†), establishing pH 3 aqueous solution as an optimum for TiO_2_|NiP. This optimum pH for TiO_2_|NiP is in agreement with the previously reported higher electrocatalytic activity of NiP in pH 3 compared to pH 4 in homogeneous solution.^20^ The pendant amines in NiP have a p*K*_a_ of approximately 3,^41^ suggesting this to be the optimum pH for proton transfer to the Ni centre, which is a key mechanistic feature of this class of catalyst.^22^

We have also tested the influence of O_2_ on the activity of TiO_2_|NiP as O_2_ tolerance is an important property for a proton reduction catalyst in water splitting.^27,42,43^ The H_2_ production activity by the TiO_2_|NiP cathode was found to be less effective in a pH 3 electrolyte solution under an atmosphere of air, compared to when purged with N_2_ as presented above. When subjected to CPE at *E*_appl_ = −0.25 V *vs.* RHE for 4 h under air, the TiO_2_|NiP retained only some activity (Fig. S7†), achieving a TON of 39 ± 16 but with a low faradaic efficiency of 7%. This significant drop in activity in the presence of oxygen corroborates a similar deactivation observed for NiP in homogeneous aqueous solution.^27^ Thus, a membrane and a two-compartment electrochemical cell are required in PEC water splitting to protect NiP from O_2_ generated during simultaneous water oxidation at the photoanode (see below).

These results establish mesoTiO_2_ as an inexpensive, easily-prepared mesoporous cathode material for immobilisation of phosphonate-bearing molecular synthetic catalysts, particularly in terms of the stability of both the material itself and the interaction between the TiO_2_ and the molecular catalyst, enabling high turnover numbers to be reached and substantial amounts of H_2_ being generated at a modest overpotential. Our approach thereby complements previous work, where Ni(ii) bis(diphosphine) complexes were attached onto electrode surfaces such as silicon^44^ and carbon-based materials.^21,45,46^ Compared to these previous approaches, TiO_2_|NiP is easier to assemble, requires a less expensive substrate, and can be studied spectroelectrochemically. Furthermore, the transparent nature of TiO_2_ to visible light and the demonstrated high activity in mild aqueous conditions make TiO_2_|NiP a suitable cathode material for combination with a photoanode to allow for full water splitting, which is difficult to achieve for cathodes operating under the less sustainable conditions used previously.^21,44,46^ The use of TiO_2_ as cathode material in this work is also of interest in the context of current work on its use as a protection layer for photocathode materials such as Cu_2_O,^7,47^ Si^48–50^ and CuInS_2_/CdS,^51^ all of which demonstrate photoelectrochemical H_2_ production in the presence of a Pt catalyst. Employment of phosphonated molecular catalysts such as NiP on such TiO_2_ layers appears as a promising approach to replace expensive noble metals.

### Hybrid oxygen-evolving photoanode

Photoanodes composed of molecular hybrid materials developed thus far largely fall into the category of n-type TiO_2_ sensitised with molecular dyes, including those based on Ru(ii),^4,52,53^ Zn(ii)^54^ and organic molecules,^19^ and a co-immobilised molecular water oxidation catalyst. Alternatives have been developed utilising visible-light-harvesting semiconductors based on Earth-abundant metal oxides.^23,55–57^ Of these, WO_3_ is notable for good stability under the mildly acidic aqueous conditions required for operation with TiO_2_|NiP,^58^ whereas photoanodes made of α-Fe_2_O_3_ and BiVO_4_ are typically studied in alkaline^57,59^ and pH neutral conditions,^56,60,61^ respectively. It has recently been shown that an immobilised molecular Fe-based catalyst could improve the otherwise poor activity and selectivity^62^ for O_2_ evolution of WO_3_ in aqueous pH 3 Na_2_SO_4_ solution.^23^ We therefore assembled a photoanode using WO_3_,^63^ and an Fe(ii) catalyst based on a phosphonic acid-modified tris(2-picolyl)amine (TPA) ligand, the unmodified triflate-coordinated analogue of which is known to perform water oxidation homogeneously at low pH in the presence of a chemical oxidant.^12^

**Scheme 2:**
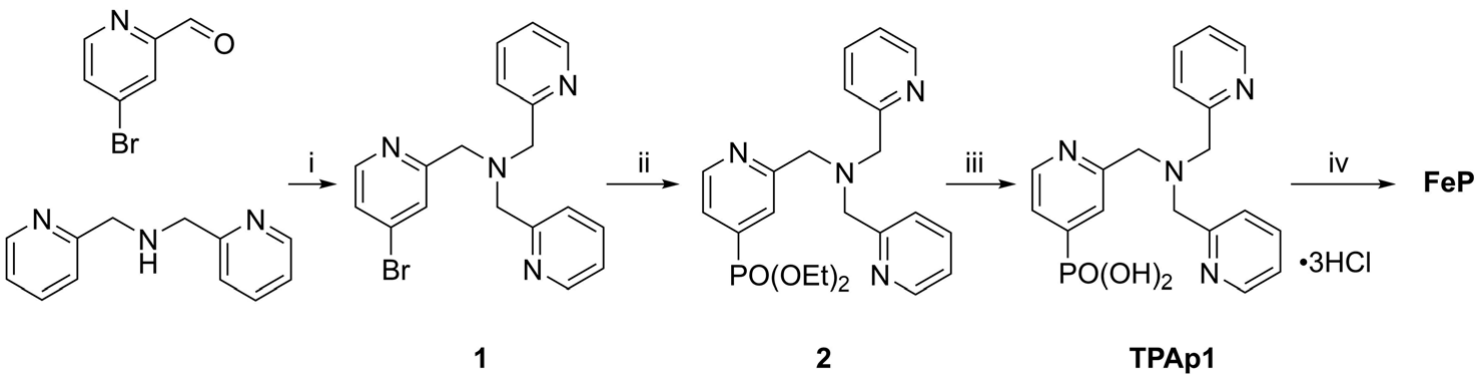
Synthetic pathway to FeP. (i) Na(AcO)_3_BH (1.2 eq.), CH_2_Cl_2_, r.t., 2 d, 66%; (ii) Pd(OAc)_2_ (4 mol%), 1,1′-bis(diphenylphosphino)ferrocene (5 mol%), Et_3_N, HPO(OEt)_2_ (1.1 eq.), 80 °C, 2 d, 67%; (iii) HCl (18% in H_2_O), reflux, 18 h, 70%; (iv) FeCl_2_, Et_3_N, CH_3_OH, r.t., 47%. Full synthetic and characterisation details can be found in the Experimental section.

Incorporation of a phosphonic acid linker group into the TPA ligand was achieved *via* a multi-step chemical synthesis shown in Scheme 2 (see Experimental section for details). Reductive amination of 4-bromopyridine-2-carboxaldehyde with bis(2-picolyl)amine resulted in the bromine-derivatised TPA compound 1. The phosphonic acid was introduced first by Pd-catalysed cross coupling^64^ to yield the phosphonate ester 2 and subsequently hydrolysed in aqueous HCl to give TPAp1·3HCl. The phosphonic acid-modified TPA ligand was coordinated to FeCl_2_ in methanol in the presence of Et_3_N to precipitate FeP (Scheme 1). FeP was synthesised in an overall yield of 15% from 4-bromo-2-pyridinecarboxaldehyde and characterised by electrospray ionisation mass spectrometry (Fig. S8†), CHN elemental analysis and IR spectroscopy.

The electrochemical response of immobilised FeP was first studied on a conducting mesoporous ITO electrode (synthesised as described in the Experimental section). Immobilisation was achieved by submersion in an FeP solution (2 mM in methanol) overnight at room temperature, and representative CVs are shown in Fig. S9.† The ITO|FeP electrodes displayed a reversible redox wave in pH 3 aqueous solution at *E*_1/2_ = 0.7 V *vs.* RHE and a linear dependence of the peak current with scan rate supports the immobilisation of FeP on the metal oxide electrode surface.

**Fig. 3:**
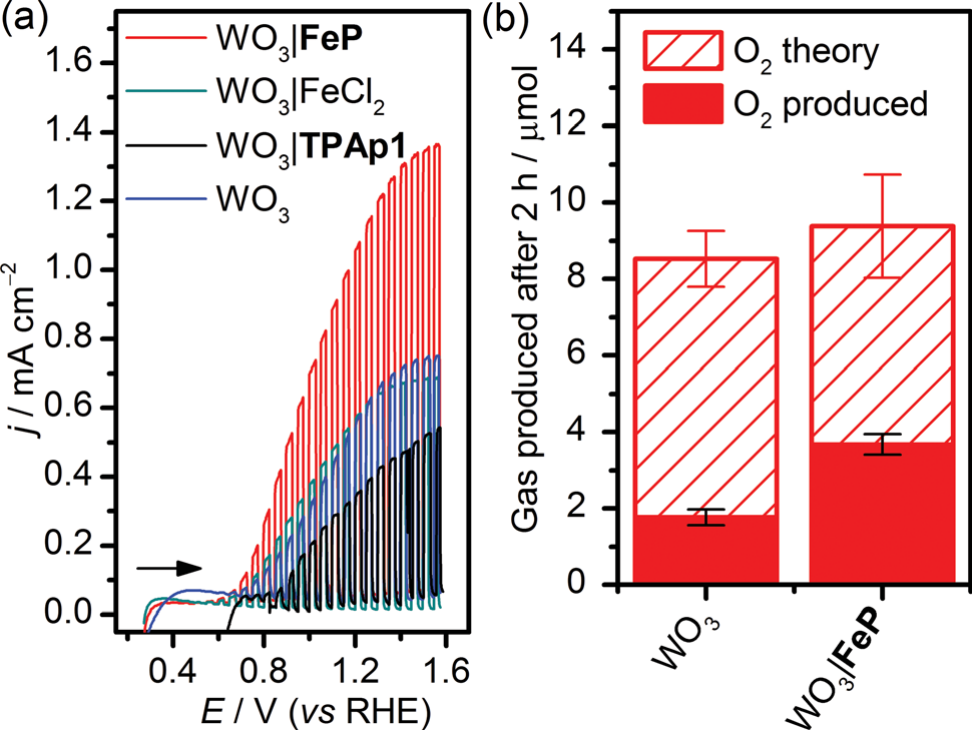
(a) LSVs under chopped illumination (AM1.5G, 0.2 W cm^−2^) of WO_3_ (blue line), WO_3_|FeP (red line), WO_3_|FeCl_2_ (green line) and WO_3_|TPAp1 at *ν* = 5 mV s^−1^. (b) Amount of O_2_ generated after two h PEC water oxidation by nanoWO_3_ with and without FeP under solar illumination at *E*_appl_ = 1.0 V *vs.* RHE. The theoretical amount of O_2_ was calculated based on 100% faradaic yield and the experimentally measured O_2_ was quantified by a fluorescence probe. Conditions: a Ag/AgCl reference electrode and a Pt mesh counter electrode in aqueous Na_2_SO_4_ (0.1 M) solution at pH 3, N_2_ atmosphere and room temperature.

WO_3_ nanosheet (nanoWO_3_) electrodes were synthesised hydrothermally onto a WO_3_ seed layer deposited on FTO as previously described (SEM image shown in Fig. S1b†).^63^ Modification of nanoWO_3_ was achieved by submersion of the electrodes in a methanol solution of FeP (2 mM) overnight at room temperature in the dark, and the UV/vis spectrum shows a small increase in absorbance between 400 and 450 nm (Fig. S10a†), consistent with the presence of FeP (Fig. S10b†). Fig. 3a shows an increased photocurrent density (*j*/mA cm^−2^) from FeP-modified WO_3_ films in comparison to unmodified films and those treated with FeCl_2_ and the TPAp1 ligand was demonstrated by linear sweep voltammetry with chopped illumination (*P* = 0.2 W cm^−2^, air mass 1.5G). This result suggests an improved photoelectrocatalytic water oxidation by the WO_3_ film modified with the molecular catalyst, but not the constituent metal salt or ligand in isolation. This result was corroborated by CPE at *E*_appl_ = 1.0 V *vs.* RHE under illumination, with the charge equivalents and amount of O_2_ produced shown in Fig. 3b. After 2 h, the WO_3_|FeP was found to produce 3.7 ± 0.4 μmol O_2_ with 40 ± 4% faradaic efficiency, compared to 1.8 ± 0.3 μmol O_2_ with 21 ± 2.1% for the unmodified electrode. The low efficiencies can be explained by competing electrolyte oxidation and incomplete oxidation of water known to occur under acidic aqueous conditions,^65^ but the increase in selectivity in the presence of the iron(ii) catalyst matched the precedent set previously.^23^

The incident photon-to-current efficiency (IPCE) of WO_3_|FeP was found to vary with the wavelength of incident monochromatic light in accordance with the UV/vis spectrum of WO_3_ (Fig. S10a†), and made efficient use of the solar spectrum at wavelengths below 450 nm. The IPCE of WO_3_|FeP reached a peak value of 53% at a wavelength of 350 nm, and corresponded to previous reports for catalyst-modified WO_3_.^66^

### Molecule-enabled PEC water splitting

**Fig. 4:**
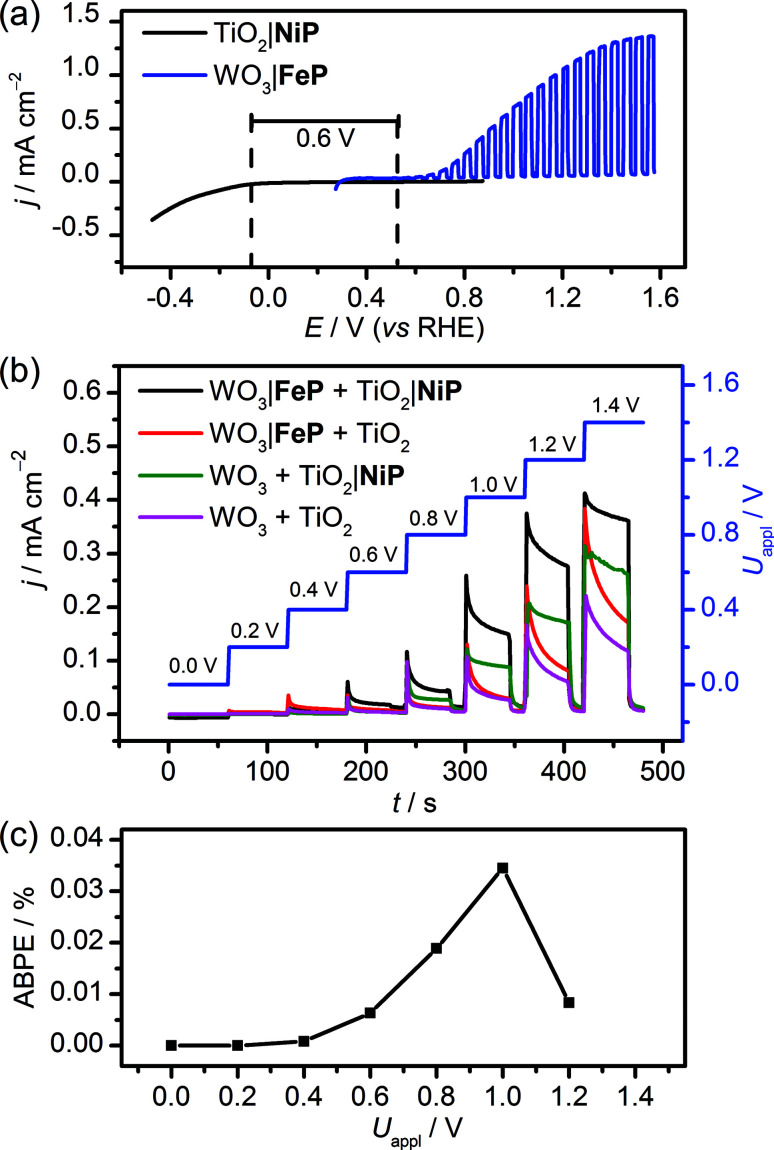
Comparison between (a) superimposed three-electrode LSVs with TiO_2_|NiP and WO_3_|FeP (*ν* = 5 mV s^−1^) and (b) two-electrode, stepped-potential PEC water splitting with TiO_2_|NiP directly wired to WO_3_|FeP (control experiments in the absence of one or both molecular catalysts are also shown). (c) Applied bias photon-to-current efficiency, ABPE for the cell comprising both catalyst-modified electrodes. Conditions: aqueous Na_2_SO_4_ (0.1 M) electrolyte solution at pH 3 and room temperature with solar light irradiation (100 mW cm^−2^ AM1.5G) using a TiO_2_|NiP cathode (geometrical surface area: 1.0 cm^2^) and a WO_3_|FeP photoanode (0.5 cm^2^).

The WO_3_|FeP photoanode displays good water oxidation activity and is compatible with the H_2_-evolving TiO_2_|NiP cathode in an aqueous pH 3 electrolyte solution. Fig. 4a shows a superposition of the three-electrode linear sweep voltammograms of the individual electrodes. The voltammograms of the individual electrodes imply that if the TiO_2_|NiP cathode and WO_3_|FeP photoanode are combined in a two-electrode PEC configuration, the onset of photocurrent should be at an applied voltage of approximately 0.6 V. This is lower than the thermodynamic potential requirement for water splitting (1.23 V) and the proposed device should have capacity for solar energy storage in the fuel H_2_.

We therefore assembled a two-electrode PEC cell, with WO_3_|FeP as the photoanode under periodic simulated solar light irradiation and TiO_2_|NiP (shielded from the illumination to avoid UV band gap excitation of TiO_2_) as the cathode, with the electrodes separated by a proton-permeable Nafion membrane to prevent gas diffusion between the cathodic and anodic compartments (Scheme 1). As predicted from the voltammetric response of the individual electrodes, a notable photocurrent was observed at *U*_appl_ = 0.6 V in the two-electrode configuration linear sweep voltammogram shown in Fig. 4b. This demonstrates energy storage across the electrodes without the need for a precious-metal containing component (catalyst and electrode material). In the absence of NiP, the photocurrent decayed quickly within each 45 s illumination period, due to initial reductive charging of the TiO_2_ (Fig. 4b). The applied bias (*U*_appl_) photon-to-current conversion efficiency (ABPE, Fig. 4c) for the PEC cell was calculated with eqn (1), with the photocurrent density (*j*/mA cm^−2^) taken after 45 s illumination at each *U*_appl_ under full simulated solar spectrum irradiation with a light intensity (*P*) of 100 mW cm^−2^.1
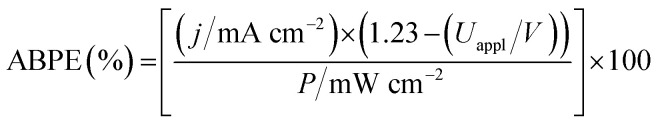


The ABPE was found to have a maximum at *U*_appl_ = 1.0 V with an ABPE = 0.035%. The low value for ABPE is due to incomplete use of the solar spectrum by WO_3_ shown in Fig. S10a† (note that the full simulated solar spectrum, not monochromatic light, was used to calculate the ABPE) and a relatively large applied bias required to achieve a significant photocurrent. Nevertheless, this efficiency is comparable to the only other efficiency reported for a fully molecular solar PEC water splitting cell with a solar-to-hydrogen (STH) efficiency of 0.05%, which required the use of precious metals. However it should be noted that STH accounts for the faradaic efficiency of H_2_ production, unlike the APBE presented here.^19^

**Fig. 5:**
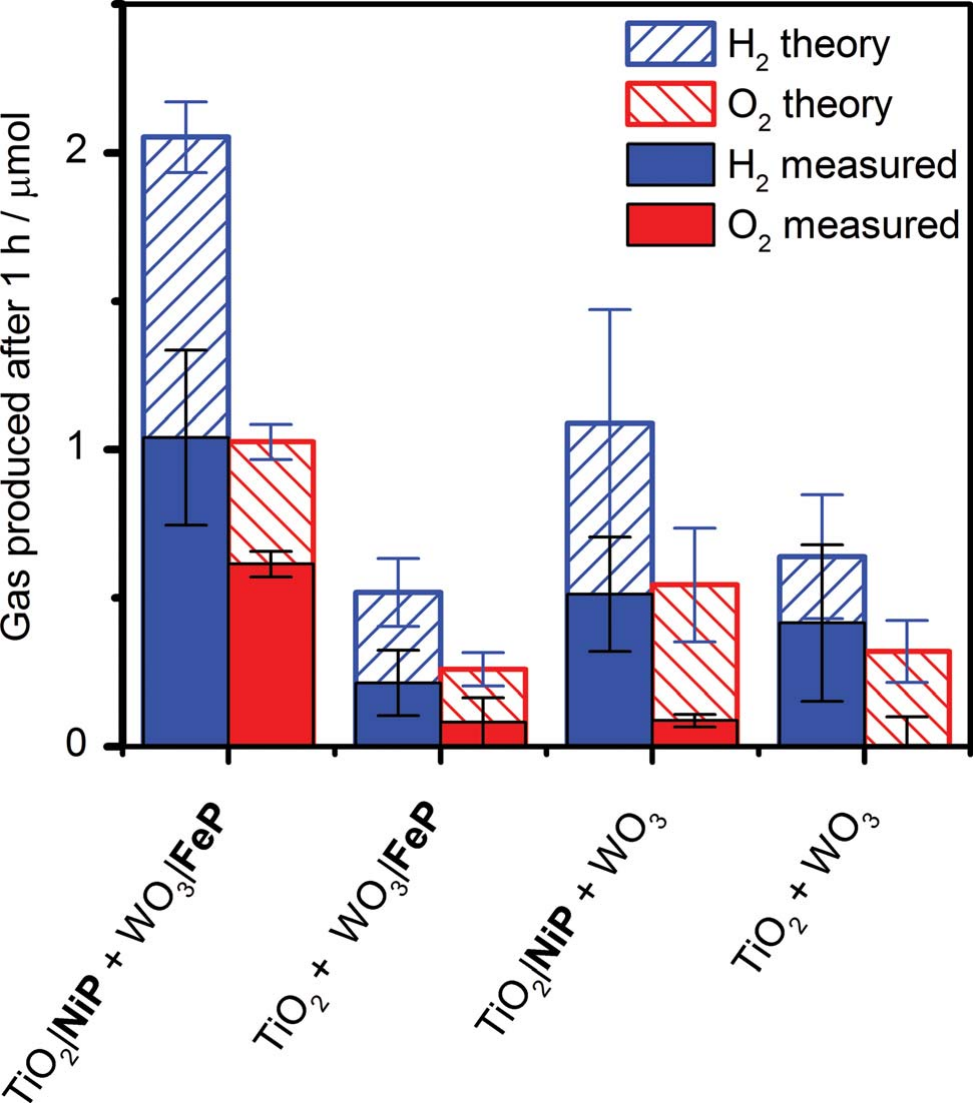
Summary of theoretical and practical H_2_ and O_2_ production by two-electrode PEC water splitting with TiO_2_|NiP cathode (geometrical surface area: 1.0 cm^2^) wired to WO_3_|FeP photoanode (0.5 cm^2^) and control experiments without one or both molecular catalysts. The theoretical amount of gaseous products were calculated based on 100% faradaic yield. Conditions: aqueous Na_2_SO_4_ (0.1 M) electrolyte solution at pH 3, N_2_ atmosphere and room temperature with an applied bias (*U*_appl_) = 1.1 V for 1 h and solar light illumination (100 mW cm^−2^, AM1.5G).

It was essential to perform extended PEC water splitting under simulated solar light illumination for an in-depth assessment of the efficacy of the molecular catalysts. An electrochemical bias of 1.1 V, less than the thermodynamic potential for water splitting, was applied, and the generated charge, amounts of O_2_ and H_2_ are summarised in Table 1 and Fig. 5. After 1 h, 0.61 ± 0.06 μmol of O_2_ were detected by a fluorescence sensor (Fig. S11†) and 1.04 ± 0.29 μmol of H_2_ were analysed by gas chromatography with faradaic efficiencies of 61 ± 5% and 53 ± 17%, respectively. The O_2_-to-H_2_ ratio was close to the expected one-to-two ratio for full water splitting. Consistent with the three-electrode experiments was the observation that in the absence of FeP, no or only traces of oxygen were detected, again demonstrating the increased selectivity offered by the molecular FeP catalyst in these conditions. The lower faradaic efficiency for H_2_ evolution compared to the three-electrode CPE Table 1 may be due to initial competitive reduction of O_2_ or other contaminants trapped within the mesoporous TiO_2_ electrode in the early stages of electrolysis, as has been observed previously for other nanostructured electrodes.^67^ In agreement, we observed a faradaic efficiency of 58 ± 13% for H_2_ generation with TiO_2_|NiP after 1 h CPE in the experiments shown in Fig. 2a.

Key performance parameters of the electrodes employed in this work^a^

**Table:** 

Three-electrode configuration^b^						
Description	*E*_appl_/V *vs.* RHE	Time/h	*n*(H_2_)/μmol	Faradaic yield (%)	*n*(O_2_)/μmol	Faradaic yield (%)
TiO_2_|NiP	−0.25	8	9.3 ± 2.1	88 ± 17		
TiO_2_	−0.25	8	0.07 ± 0.03	5.6 ± 1.7		
TiO_2_|NiP^c^	−0.25	4	0.58 ± 0.24	7.4 ± 4.5		
WO_3_|FeP	1.0	2			3.7 ± 0.4	40 ± 4
WO_3_	1.0	2			1.8 ± 0.3	21 ± 2.1

a Conditions: aqueous Na_2_SO_4_ (0.1 M) solution at pH 3 in a two compartment (photo)electrochemical cell at room temperature. Unless otherwise stated, the cell purged with N_2_ before each experiment and CH_4_ (2%) was present as internal standard for H_2_ quantification by GC. The WO_3_ photoanode was illuminated with solar light (AM1.5G) at 0.2 W cm^−2^ in the three-electrode experiments and 0.1 W cm^−2^ in two-electrode PEC water splitting. The TiO_2_ cathode was shielded from illumination.

b A Pt counter electrode and a Ag/AgCl reference electrode were employed.

c Performed under air with CH_4_ as external standard.

d Below the limit of detection.

**Table:** 

Two-electrode PEC water splitting						
Description	*U*_appl_/V	Time/h	*n*(H_2_)/μmol	Faradaic yield (%)	*n*(O_2_)/μmol	Faradaic yield (%)
TiO_2_|NiP + WO_3_|FeP	1.1	1	1.04 ± 0.29	53 ± 17	0.61 ± 0.06	61 ± 6
TiO_2_|NiP + WO_3_	1.1	1	0.51 ± 0.19	47 ± 14	0.09 ± 0.02	18 ± 6
TiO_2_ + WO_3_|FeP	1.1	1	0.21 ± 0.1	38 ± 13	0.08 ± 0.08	26 ± 26
TiO_2_ + WO_3_	1.1	1	0.41 ± 0.26	60 ± 18	—d	—
TiO_2_|NiP + WO_3_|FeP	1.23	1	2.3 ± 0.4	83 ± 16	0.82 ± 0.10	60 ± 20

Without NiP, low photocurrents and consequently lower gaseous products were detected, showing that the molecular catalyst is required to perform proton reduction at a sufficiently low overpotential to be coupled to water oxidation in this system. We also performed two-electrode PEC water splitting of the molecular catalyst-modified electrodes at zero-energy storage (*U*_appl_ = 1.23 V *vs.* RHE) for 1 h, with the results summarised in Table 1. We observed a higher H_2_ (2.3 ± 0.4 μmol, 83 ± 16% faradaic yield) and O_2_ (0.82 ± 0.10 μmol, 60 ± 20% faradaic yield) evolution activity than at *U*_appl_ = 1.1 V. These results demonstrate that the molecular catalysts based on Earth-abundant transition metals are also capable of driving water splitting at a higher rate if better electrode materials become available. A tandem PEC device with a suitable photocathode modified with NiP wired to WO_3_|FeP would dramatically reduce the applied bias and result in higher photocurrents and ABPE in the future.^7,68^

### Molecular integrity of catalysts in PEC water splitting

**Fig. 6:**
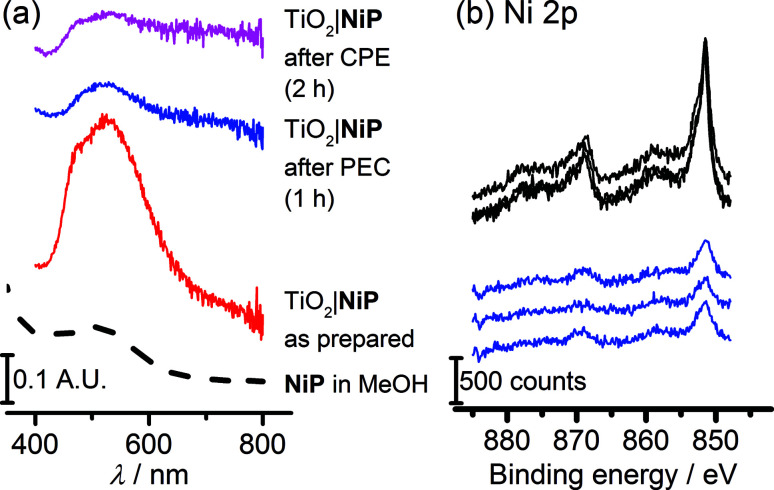
(a) Diffuse-reflectance UV-vis absorption spectra of NiP-modified TiO_2_ electrodes as prepared (red line), after 1 h PEC electrolysis at *U*_appl_ = 1.1 V, and after 2 h CPE at *E*_appl_ = −0.33 V *vs.* RHE, with a MeOH solution UV-vis spectrum as a reference (dashed line). (b) X-ray photoelectron spectra in the Ni 2p region of TiO_2_|NiP and electrodes before (black line) and after (blue line) 1 h PEC electrolysis at *U*_appl_ = 1.1 V with WO_3_|FeP.

The study of molecular catalysts under strongly reductive and oxidative conditions requires an assessment of their integrity during catalysis.^69^ For instance, Fe-based catalysts related to FeP are known to remain molecular during water oxidation in homogeneous aqueous acidic conditions, but to oxidise to catalytically-active iron oxide nanoparticles under basic conditions.^70^ Although Ni-based molecular compounds have also been reported to decompose to catalytically-active Ni-containing nanoparticles under reductive conditions,^71^ this has not been observed for NiP in solution or in suspensions with semiconducting nanoparticles.^14,20,26,27^Fig. 6a shows diffuse reflectance UV-vis spectra of the NiP-modified mesoTiO_2_ electrode. Before electrolysis, the electrodes were purple in colour and showed a broad band at *λ*_max_ = 520 nm in the UV-vis spectrum of NiP. The purple colour with the band at *λ*_max_ = 520 nm were qualitatively retained following three-electrode CPE for 2 h and two-electrode solar water electrolysis coupled to WO_3_|FeP for 1 h, indicating that molecular NiP remained on mesoTiO_2_.

X-ray photoelectron spectroscopy (XPS) on both modified electrodes before and after running 1 h PEC water splitting at an applied bias of 1.1 V were performed to analyse the possible formation of metal-containing deposits. The Ni(2p) or Fe(2p) : N(1s) : P(2p) ratios, of approximately 1 : 4 : 8 for TiO_2_|NiP and 1 : 4 : 1 for WO_3_|FeP, before two-electrode PEC water splitting were as expected (Scheme 1 and Table S1†). In agreement with previous work, for WO_3_|FeP, no Fe peak was observed after PEC water splitting (Fig. S12†), suggesting that no iron-based deposit was formed on the electrode. Instead it is postulated that the iron(ii) catalyst slowly desorbs from the electrode surface during photoelectrocatalysis,^23^ possibly due to the presence of only one phosphonate anchor group on FeP. Therefore, the limiting electrode in this PEC water splitting cell is the FeP-modified WO_3_ photoanode.

Peaks corresponding to Ni(2p), N(1s) and P(2p) were retained after catalysis (Fig. 6b and S13†), and remained in the same ratio before and after PEC water splitting for TiO_2_|NiP. Fig. 6b and the peak positions in Table S2† demonstrate that the Ni(2p) peak binding energies at 851.5 eV and 869 eV remain unchanged during PEC water splitting, and no new peaks were observed that could correspond to a new Ni-based species. The combination of the unchanged elemental ratios, unaltered peak positions in XPS and diffuse reflectance UV-vis data provides strong evidence that the molecular structure of NiP remains intact during electrolysis immobilised on mesoTiO_2_ electrodes. This result emphasises the suitability of molecular electrocatalyst design even when working on heterogeneous catalytic systems.

## Conclusions

We have presented a hybrid proton reduction cathode based on a phosphonated Ni(ii) bis(diphosphine) molecular catalyst and mesostructured TiO_2_, which is normally considered an insulator in the absence of UV irradiation and therefore of limited use in electrocatalysis. We observed, from complementary cyclic voltammetry and spectroelectrochemistry, that at mildly reducing potentials electrons are transferred first to the CB of TiO_2_, and then to NiP for proton reduction catalysis. During long term controlled-potential electrolysis, sustained H_2_ production was observed at an applied potential of *E*_appl_ = −0.25 V *vs.* RHE, with a NiP-based turnover number of 600 achieved after 8 hours. Characterisation and quantification of the catalyst after electrolysis revealed not only that the molecular structure was intact, but also 75% of the NiP initially present remained on the electrode surface. The modified TiO_2_ performed considerably better than an analogous mesoporous ITO electrode, which underwent degradation under reducing conditions. Therefore, the TiO_2_|NiP cathode exhibited excellent stability in terms of the material itself, the molecular structure of the catalyst, and the attachment between the two in mildly acidic aqueous conditions. These results establish TiO_2_|NiP as not only an effective H_2_ evolving electrode in its own right, but also suggest potential as a catalytically-active protection layer for photocathodes due to its ease of preparation, transparency to visible light, stability and high activity at a modest overpotential.

We have demonstrated the utility of this cathode by constructing a precious-metal-free two-compartment PEC cell for full water splitting with molecular catalysts crucial to the performance of both the photoanode and the cathode for the first time. Under solar illumination and an applied bias of *U*_appl_ = 1.1 V, below the thermodynamic potential for water splitting, an approximately 2 : 1 ratio of H_2_ : O_2_ was only obtained in the presence of FeP and NiP. Essential to this achievement was the development of hybrid electrodes operable under the same mild conditions. We have also presented a number of experiments showing consistent evidence that the molecular structure of NiP remained intact after the photoelectrolysis experiments, a result which emphasises the importance in molecular catalyst design in the development of molecule/material hybrids. These results demonstrate that if mild conditions are used, molecular catalysts can remain stable and effective under catalytic conditions. The immobilisation approach and characterisation techniques reported here provide a promising framework for the future systematic study of the activity and stability of a variety of molecular catalysts in environmentally-benign aqueous solution.

## Experimental section

### Materials and methods

All starting materials were obtained from commercial sources and used without further purification, unless otherwise stated. ITO nanopowder (<50 nm particle size) was obtained from Sigma-Aldrich and P25 TiO_2_ (8 : 2 anatase : rutile, 20 nm average particle size) from Evonik Industries. NiP^20^ and 4-bromo-2-pyridinecarboxaldehyde^72^ were synthesised following literature procedures. Dry solvents were dried and distilled under N_2_ prior to use, or in the case of methanol purchased as dry solvent and stored over molecular sieves. Solvent mixtures are reported as vol : vol ratios. Standard Schlenk line techniques were used where required. ^1^H, ^13^C and ^31^P NMR measurements were performed on a Bruker DPX400 spectrometer. Mass spectrometry measurements were performed on a Waters Micromass Quattro LS ESI or ThermoScientific Orbitrap Classic instrument (calculated and experimental isotope patterns were compared). SEM was performed on a FEI Phillips XL30 field emission gun SEM instrument. XPS was performed by the Nexus facility at the University of Newcastle on a K-Alpha (Thermo Scientific, East Grinstead, UK) spectrometer utilising a monochromatic Al-Kα X-ray source (1486.6 eV, 400 μm spot size, 36 W). Survey spectra were collected with a pass energy of 200 eV and 3 sweeps, while high resolution spectra were collected at a pass energy of 40 eV with 10 sweeps. Elemental analysis was carried out by the University of Cambridge Microanalysis Service using a Perkin-Elmer 240 Elemental Analyser. UV-vis absorption spectroscopy was performed using a Varian Cary 50 spectrophotometer. Where stated, a diffuse reflectance accessory for the spectrophotometer was used, with a Spectralon reference as a background. UV-vis spectra (reflectance mode) of WO_3_ were recorded on an Edinburgh Instruments FS5 spectrofluorometer equipped with an integrating sphere by running a synchronous scan (*λ*_ex_ = *λ*_em_) and subtracting a scan of the sphere background. ATR-FTIR measurements were performed on a Nicolet iS50 FTIR spectrometer.

### Synthesis and characterisation

#### Bis(2-methylpyridyl)(4-bromo-2-methylpyridyl)amine (1)

This compound was synthesised by adaptation of a literature procedure for tris(2-pyridylmethyl)amine.^72^ In a pre-dried Schlenk flask, sodium tri(acetoxy)borohydride (1.7 g, 6.6 mmol) was dried *in vacuo*. Dry CH_2_Cl_2_ (50 mL) was added, followed by 4-bromo-2-pyridinecarboxaldehyde (1.0 g, 5.6 mmol) and di-(2-picolylamine) (1.0 mL, 4.7 mmol). The mixture was degassed by three freeze–pump–thaw cycles, purged with N_2_ and stirred under N_2_ at room temperature for 2 d. Saturated aqueous NaHCO_3_ (20 mL) was added, and the biphasic mixture stirred for 1 h. The crude product was extracted in CH_2_Cl_2_ (3 × 25 mL), washed with brine (20 mL), dried over MgSO_4_ and the solvent removed *in vacuo*. Purification was achieved by flash column chromatography on silica (pre-deactivated with Et_3_N), eluting in CH_2_Cl_2_ : Et_3_N (98 : 2) to yield 1 as a pale yellow oil (yield: 1.1 g, 66%). HR-MS calc. for [C_18_H_18_BrN_4_]^+^: 369.0709, found: 369.0724 (100% peak); ^1^H-NMR (400 MHz, CDCl_3_) *δ*/ppm: 8.58 (d, *J* = 4.1 Hz, 2H), 8.36 (d, *J* = 5.3 Hz, 1H), 7.80 (d, *J* = 1.5 Hz, 1H), 7.70 (td, *J* = 7.7, 1.6 Hz, 2H), 7.56 (d, *J* = 7.6 Hz, 2H), 7.34 (dd, *J* = 5.3, 1.8 Hz, 1H), 7.18 (dd, *J* = 6.6, 5.1 Hz, 2H), 3.93 (s, 4H, C*H*_2_), 3.91 (s, 2H, C*H*_2_); ^13^C NMR (101 MHz, CDCl_3_) *δ*/ppm 149.82, 148.46, 137.52, 136.93, 133.62, 126.67, 125.82, 123.75, 122.74, 120.88, 60.40, 59.33.

#### Bis(2-methylpyridyl)(4-(diethylphosphonate)-2-methylpyridyl)amine (2)

In a pre-dried Schlenk flask with a Teflon screw-cap, palladium(ii) acetate (25 mg, 0.11 mmol) and 1,1′-bis(diphenylphosphino)ferrocene (dppf, 75 mg, 0.14 mmol) were dried *in vacuo*. In a separate pre-dried flask, 1 (1.0 g, 2.7 mmol) was added and the flask evacuated and purged with N_2_ three times. The compound was dissolved in dry acetonitrile (6 mL) and added to the reaction flask under N_2_. Diethyl phosphite (0.40 mL, 2.9 mmol) and Et_3_N (1 mL) were added and the mixture was degassed by three freeze–pump–thaw cycles. The flask was heated to 70 °C under N_2_, sealed, and stirred at 70 °C for 2 d. The reaction mixture was cooled, the solvent removed *in vacuo*, and the product purified by flash column chromatography on silica (pre-deactivated with Et_3_N), eluting with CH_2_Cl_2_ : Et_3_N : CH_3_OH (97.5 : 2 : 0.5) to yield 2 as a yellow oil that discoloured quickly and solidified on standing (yield: 0.78 g, 67%). HR-MS calc. for [C_22_H_28_N_4_O_3_P]^+^: 427.1894, found 427.1875 (100% peak); ^1^H-NMR (400 MHz, CDCl_3_) *δ*/ppm: 8.68 (t, *J* = 5.0 Hz, 1H), 8.53 (d, *J* = 4.0 Hz, 2H), 7.96 (d, *J* = 14.1 Hz, 1H), 7.67 (td, *J* = 7.5, 1.0 Hz, 2H), 7.58 (d, *J* = 8.0 Hz, 2H), 7.50 (dd, *J* = 13.1, 4.0 Hz, 1H), 7.15 (dd, *J* = 6.3, 5.3 Hz, 2H), 4.06–4.24 (m, 4H), 3.97 (s, 2H), 3.90 (s, 4H), 1.34 (t, *J* = 7.0 Hz, 6H); ^13^C (101 MHz, CDCl_3_) *δ*/ppm: 160.74 (d, *J* = 12.0 Hz), 159.45 (s), 149.78 (d, *J* = 12.8 Hz), 149.48 (s), 138.16 (d, *J* = 187.7 Hz), 136.86 (s), 124.92 (d, *J* = 8.8 Hz), 123.83 (d, *J* = 8.8 Hz), 123.39 (s), 122.47 (s), 63.07 (d, *J* = 5.6 Hz), 60.60 (s), 60.37 (s), 16.72 (d, *J* = 6.4 Hz) ^31^P-NMR (162 MHz, CDCl_3_) *δ*/ppm: −16.31; IR *ν* = 1250 cm^−1^ (P=O).

#### Bis(2-methylpyridyl)(4-phosphonic acid-2-methylpyridyl)amine hydrochloride (TPAp1·3HCl)

Compound 2 (0.5 g, 1.2 mmol) was dissolved in 18% aqueous HCl (4 mL) and refluxed for 18 h. The reaction mixture was cooled to r.t. and the solvent was removed *in vacuo* and the brown oily crude dissolved in CH_3_OH. Precipitation with EtOAc followed by filtration and washing with EtOAc yielded TPAp1·3HCl as a hygroscopic off-white powder (yield: 0.40 g, 70%). MS calc. for [C_18_H_20_N_4_O_3_P]^+^: 371.12 (100.0%), found: 371.0 (100%); ^1^H-NMR (400 MHz, D_2_O) *δ*/ppm: 8.65 (d, *J* = 5.6 Hz, 2H), 8.60 (br. s., 1H), 8.42 (t, *J* = 7.9 Hz, 2H), 7.99 (m, 3H), 7.86 (m, 3H), 4.35 (s, 4H), 4.27 (br. s., 2H, *CH*_2_); ^13^C-NMR (101 MHz, D_2_O) *δ*/ppm: 151.42 (d, *J* = 12.1 Hz), 151.00 (s), 147.52 (s), 144.34 (d, *J* = 321.6 Hz), 142.09 (d, *J* = 12.1 Hz), 141.91 (s), 127.85 (d, *J* = 9.5 Hz), 127.53 (s), 127.16 (d, *J* = 8.7 Hz), 126.78 (s), 56.37 (s); ^31^P-NMR (162 MHz, D_2_O) *δ*/ppm: −4.36; IR *ν* = 1170 cm^−1^ (P=O); EA calc. for C_18_H_26_Cl_3_N_4_O_5_P (TPAp1·3HCl·2H_2_O) C, 41.92; H, 5.08; N, 10.86; P, 6.01, found C 42.51, H 5.01, N 10.42, P 5.81.

#### [FeCl(MeO)(TPAp1)] (FeP)

In a pre-dried Schlenk flask, TPAp1·3HCl (40 mg, 0.084 mmol) was dissolved in dry CH_3_OH under N_2_. Dry Et_3_N (50 μL, 0.33 mmol) was added, and the solution stirred under for 30 min. This solution was added to a degassed solution of anhydrous FeCl_2_ (11 mg, 0.084 mmol) in CH_3_OH (2 mL). The solution was freeze–pump–thaw degassed three times, and stirred under N_2_ for 2 h. The solvents were removed *in vacuo*, CH_3_CN (4 mL) was added, and the suspension stirred under N_2_ for 12 h to dissolve the Et_3_NHCl. The suspension was filtered off and the precipitate dried *in vacuo* to give FeP as a red powder (yield: 20 mg, 47%). MS calc. for [Fe(TPAp1)Cl]^+^: *m*/*z*: 461.022 (100.0%), found 460.97 (100%); EA calc. for C_19_H_23_ClFeN_4_O_3_P [TPAp1FeCl(OMe)] C, 46.32; H, 4.50; N, 11.37; found C, 46.02; H, 4.85 N, 11.60; IR *ν* = 1170 cm^−1^ (P=O).

### Preparation of nanostructured metal oxide electrodes

Prior to preparation of mesoporous electrodes, glass slides coated with indium-doped tin oxide (ITO) for mesoITO or fluorine-doped tin oxide (FTO) for mesoTiO_2_ of dimensions 3.0 cm × 1.0 cm were cleaned by heating at 70 °C in a 5 : 1 : 1 solution of H_2_O : H_2_O_2_ (30% aq.) : NH_4_OH (conc. aq.) for 30 min, followed by rinsing with H_2_O and drying at 180 °C for 1 h. Suspensions of ITO (20% by weight of ITO in a 5 M acetic acid solution in ethanol) and TiO_2_ nanoparticles (100 mg TiO_2_ and 50 mg poly(ethylene glycol) in approximately 1 mL ethanol) were applied to the transparent conducting oxide-coated glass slides using the doctor blading method using a Scotch tape mask with aperture dimensions of either a 6 mm diameter circle (for cyclic voltammetry) or a 0.7 cm × 1.5 cm rectangle (for (photo)electrolysis). The slides were then annealed at 450 °C for 0.5 h (mesoTiO_2_) or at 400 °C for 1 h (mesoITO).

The mesoTiO_2_ and mesoITO electrodes were cleaned and dried with ammonia/hydrogen peroxide (see above) and/or using a BioForce UV/ozone cleaner. Immobilisation was achieved by submersion of the electrodes in a 0.5 mM solution of NiP in methanol for 18 h. The coverage of NiP was quantified by submersion of the modified electrodes in a NaOH_(aq)_ solution (0.1 M) for 30 min, followed by UV-vis spectroscopy of the resultant solution and comparison with calibration data at *λ* = 257 and 300 nm.

WO_3_ electrodes were prepared as previously described by hydrothermal synthesis,^63^ and were characterised by SEM to reveal a nanosheet morphology (Fig. S1b†). Electrode areas were masked to 1.5 cm^2^ for three-electrode measurements and 0.5 cm^2^ for two-electrode measurements.

### Electrochemical methods

All electrochemical measurements were performed on Ivium Technologies CompactStat or IviumStat, or PalmSens EmStat or MultiEmStat^3+^ potentiostats. All electrochemical measurements were performed in a Na_2_SO_4_ (0.1 M) aqueous solution, adjusted to the desired pH by titration with H_2_SO_4_ at room temperature and purged with N_2_ unless otherwise stated. All electrochemical experiments on the individual TiO_2_|NiP cathode or WO_3_|FeP photoanode were performed in a three-electrode configuration with a Pt counter electrode and a Ag/AgCl/KCl_(sat)_ reference electrode, whereas full water splitting CPE experiments were performed in a two-electrode setup with TiO_2_|NiP directly (shielded from illumination) connected to WO_3_|FeP in a two-compartment airtight cell. Irradiation was provided through a quartz illumination window and the cathode was separated from the anode by a Nafion membrane. Simulated solar irradiation was provided by a Newport solar light simulator with an AM1.5G filter and a water IR filter. TiO_2_ was shielded from light to avoid UV band gap excitation. Light intensity was measured using an International Light Technologies 1400 photometer. IPCE measurements were performed using monochromatic light provided by a LOT 300 W Xe lamp equipped with a MSH300 monochromator (FWHM = 5 nm). IPCE was calculated using eqn (2).2
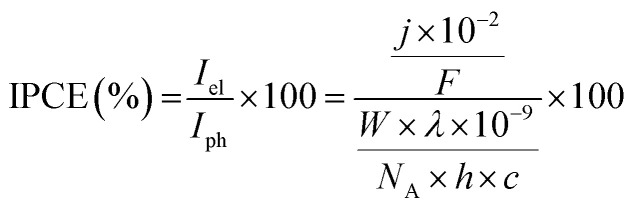
where *I*_el_ is the electron flux of the external circuit (mol m^−2^ s^−1^), *I*_ph_ is the incident photon flux (mol m^−2^ s^−1^), *j* is the measured photocurrent density (μA cm^−2^), *F* is the Faraday constant (96 484 A s mol^−1^), *λ* is the wavelength of light (nm), *W* is the incident power of the monochromated light (W m^−2^), *N*_A_ is Avogadro's number (6.022 × 10^23^ mol^−1^), *h* is Planck's constant (6.626 × 10^−34^ J s) and *c* is the speed of light (2.998 × 10^8^ m s^−1^).

Resistance between electrodes in the two compartments was found to be <100 Ω by electrochemical impedance spectroscopy and the reported voltages are not corrected for cell resistance. The experiments were performed at room temperature, and the cell was purged with an inert gas before each experiment. All reduction potentials from the three-electrode experiments are reported against RHE using eqn (3).

3 *E*_RHE_/V = *E*_Ag/AgCl_/V + 0.197 + 0.059 × pH

Spectroelectrochemical measurements were performed in a borosilicate glass one-compartment electrochemical cell purged with N_2_ in the beampath of a Varian Cary 50 spectrophotometer.

### Quantification of gaseous products

Oxygen detection was performed using an Ocean Optics NeoFox phase fluorimeter equipped with a FOSPOR probe calibrated in the 0–3% O_2_ regime using a mass-flow controller. The chamber containing the oxygen probe (in the headspace, inserted through a rubber septum) was degassed by purging the solution with N_2_ for 30 min. Background O_2_ was then recorded for 30 min, before commencement of the photoelectrolysis experiment. Finally, a further 30 min background was run after ceasing illumination and the background levels of O_2_ were subtracted. The total amount of O_2_ produced was obtained using the ideal gas law for O_2_ in the headspace, and Henry's law for dissolved O_2_.

For H_2_ quantification, the cell was purged with N_2_/CH_4_ (2%) for 10 min prior electrochemical experiments. The H_2_ produced was quantified by an Agilent 7890A gas chromatograph, with CH_4_ as an internal standard. For experiments performed under air, CH_4_ was used as an external standard. Gas detection experiments were repeated 3 times and values given as an average of the three ± standard deviation.

## Supplementary Material

SC-007-C5SC04863J-s001
