# Supplementary material for: Precious-metal free photoelectrochemical water splitting with immobilised molecular Ni and Fe redox catalysts
Source: Chem Sci. 2016 Feb 12;7(7):4024–35. doi: 10.1039/c5sc04863j (PMC6013811; doi:10.1039/c5sc04863j)
Supplement: SC-007-C5SC04863J-s001 [file SC-007-C5SC04863J-s001.pdf]

Electronic Supplementary Material (ESI) for Chemical Science.
This journal is © The Royal Society of Chemistry 2016

Electronic Supplementary Information

Precious-metal free photoelectrochemical water splitting with immobilised molecular Ni and Fe redox catalysts

Timothy E. Rosser[a], Manuela A. Gross[a], Yi-Hsuan Lai[a] and Erwin Reisner[a,*]

[a]*Christian Doppler Laboratory for Sustainable SynGas Chemistry, Department of Chemistry, University of Cambridge, Lensfield Road, Cambridge CB1 2EW, U.K.*

*Corresponding author: *reisner@ch.cam.ac.uk*

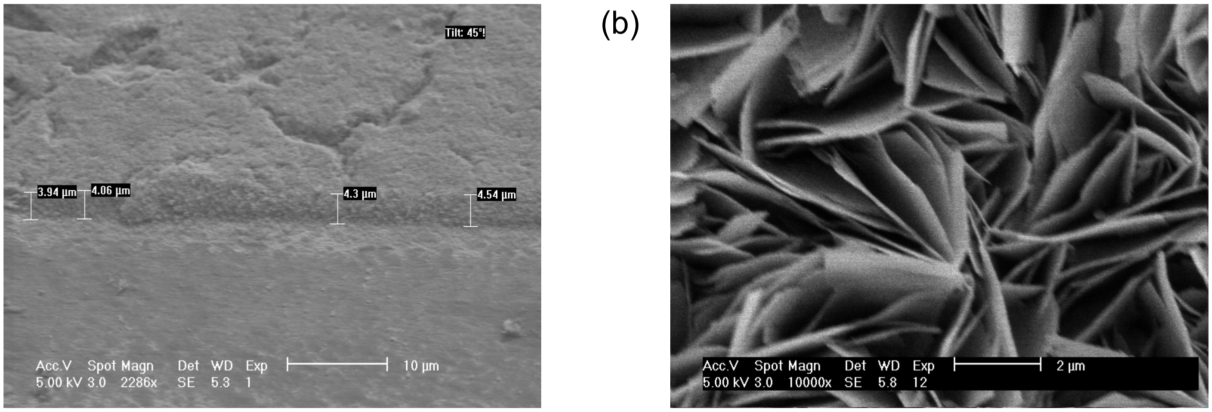

Figure S1. SEM images of (a) mesoporous TiO_2 and (b) WO_3 nanosheets.

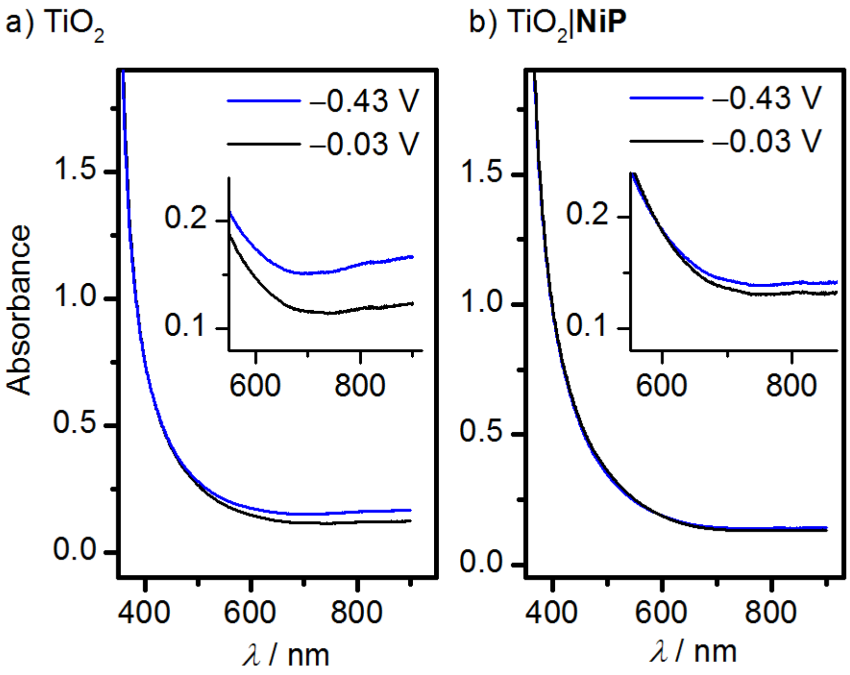

Figure S2. UV-vis spectra of (a) an unmodified and (b) a **NiP**-modified mesoTiO_2 electrode at E_{appl} = –0.43 V and –0.03 V *vs* RHE in an aqueous Na_2SO_4 solution (0.1 M) at pH 3.

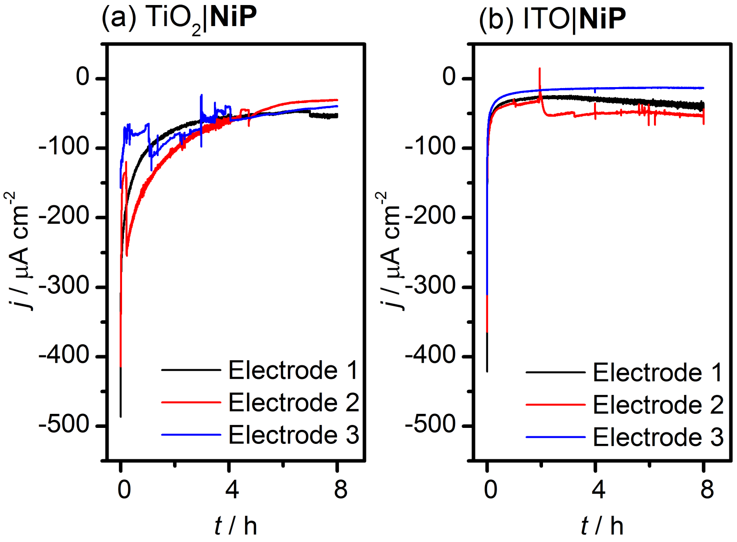

Figure S3. Chronoamperograms of (a) TiO_2|**NiP** and (b) ITO|**NiP** at E_{appl} = –0.25 V *vs* RHE, using a freshly-prepared electrode for each experiment. Conditions: Aqueous Na_2SO_4 solution (0.1 M) at pH 3 with a Ag/AgCl reference electrode and a Pt mesh counter electrode.

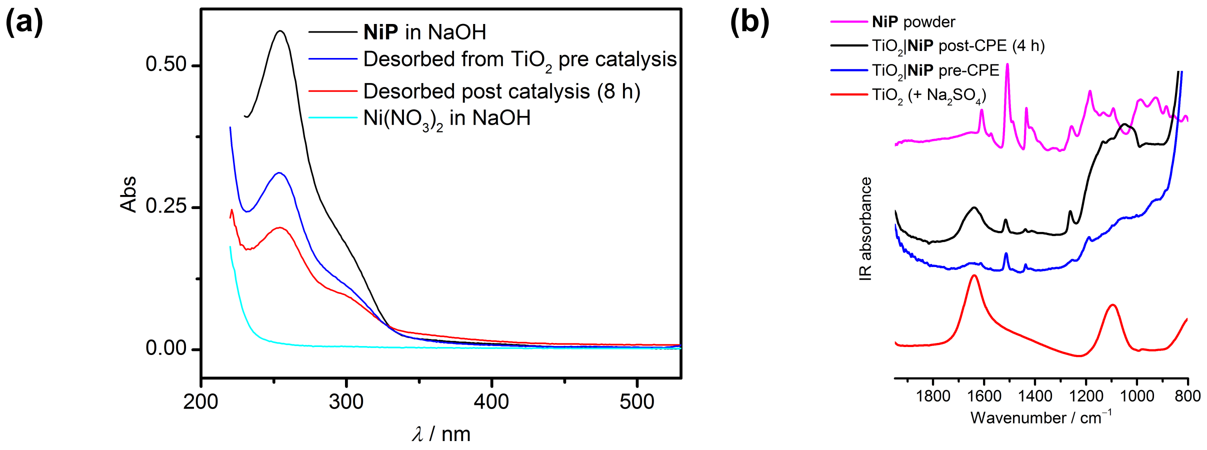

Figure S4. (a) UV-vis spectra of an aqueous NaOH solution (0.1 M) obtained after treating **NiP**-modified mesoTiO_2 electrodes before and after CPE for 8 h. Reference spectra of **NiP** (13 µM) and $Ni(NO_3)_2$ (13 µM) dissolved in NaOH (0.1 M) is shown for comparison. The spectrum of **NiP** is significantly different to that of $Ni(NO_3)_2$ in NaOH, suggesting that this is not just evidence of unassigned Ni on the electrode surface. (b) ATR-IR spectroscopy of **NiP**-modified mesoTiO_2 electrodes before and after 4 h CPE. Reference spectra of **NiP** and TiO_2 treated with 0.1 M Na_2SO_4 are also shown. CPE conditions: E_{app} = –0.25 V vs RHE, 0.1 M Na_2SO_4 (pH 3), Pt CE, Ag/AgCl RE.

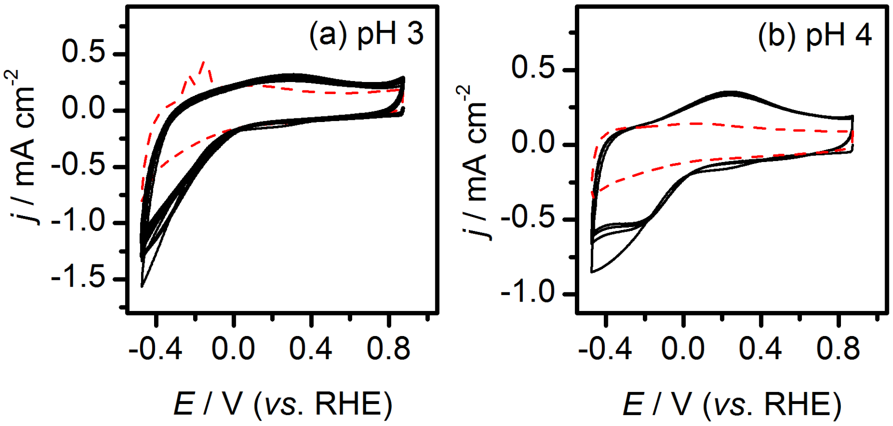

Figure S5. CVs of **NiP**-modified (solid traces) and unmodified mesoITO (dashed traces) at ν = 100 mV s^{-1}. Conditions: Aqueous Na_2SO_4 (0.1 M) solution at pH 3 and pH 4 with a Ag/AgCl reference electrode and a Pt mesh counter electrode. Oxidative waves in the CV at pH 3 stem from the re-oxidation of degraded ITO.

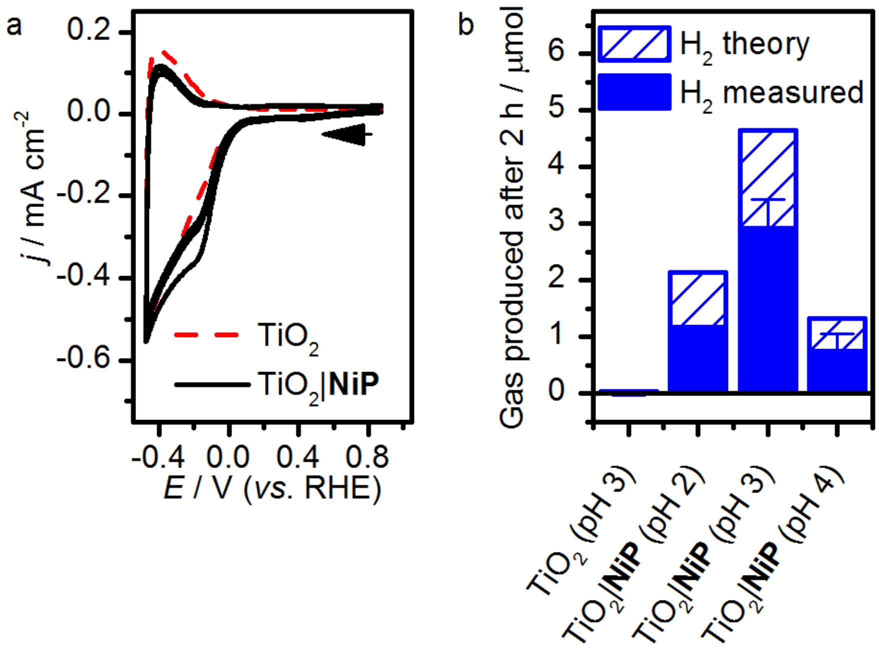

Figure S6. (a) CV of **NiP**-modified (solid trace) and unmodified (dashed trace) mesoTiO_2 in an aqueous Na_2SO_4 solution at pH 4 (ν = 100 mV s^{-1}). The arrow indicates the initial scan direction. Reductive wave at –0.2 V due to **NiP** reduction (b) Theoretical and measured H_2 after 2 h CPE of **NiP**-modified and unmodified electrodes at E_{app} = –0.33 V *vs* RHE at pH 2, 3 and 4 (0.1 M Na_2SO_4).

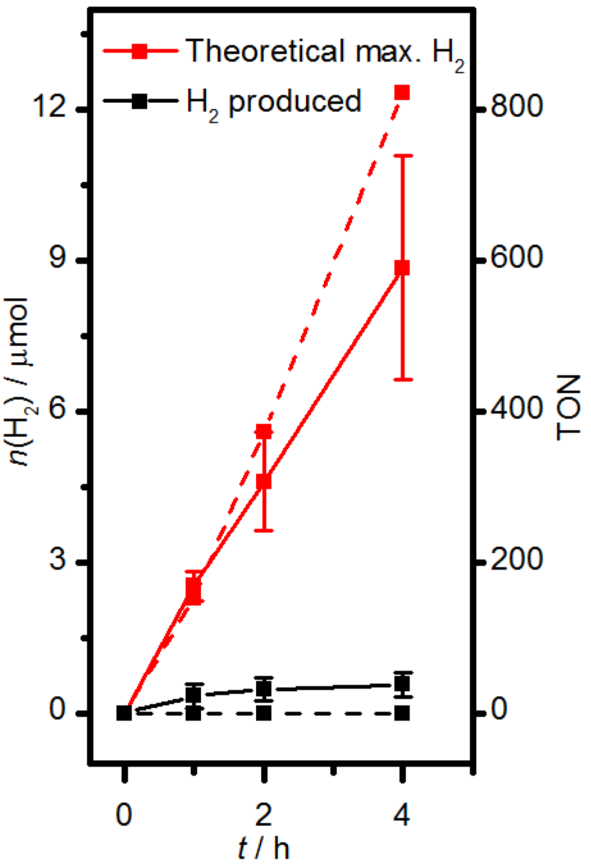

Figure S7. Theoretical (red) and measured (black) amount of H_2 after 4 h CPE with TiO_2|**NiP** (solid line) and TiO_2 (dashed line) at E_{appl} = –0.25 V *vs* RHE under air. Conditions: Aqueous Na_2SO_4 solution (0.1 M) at pH 3 with a Ag/AgCl reference electrode and a Pt mesh counter electrode.

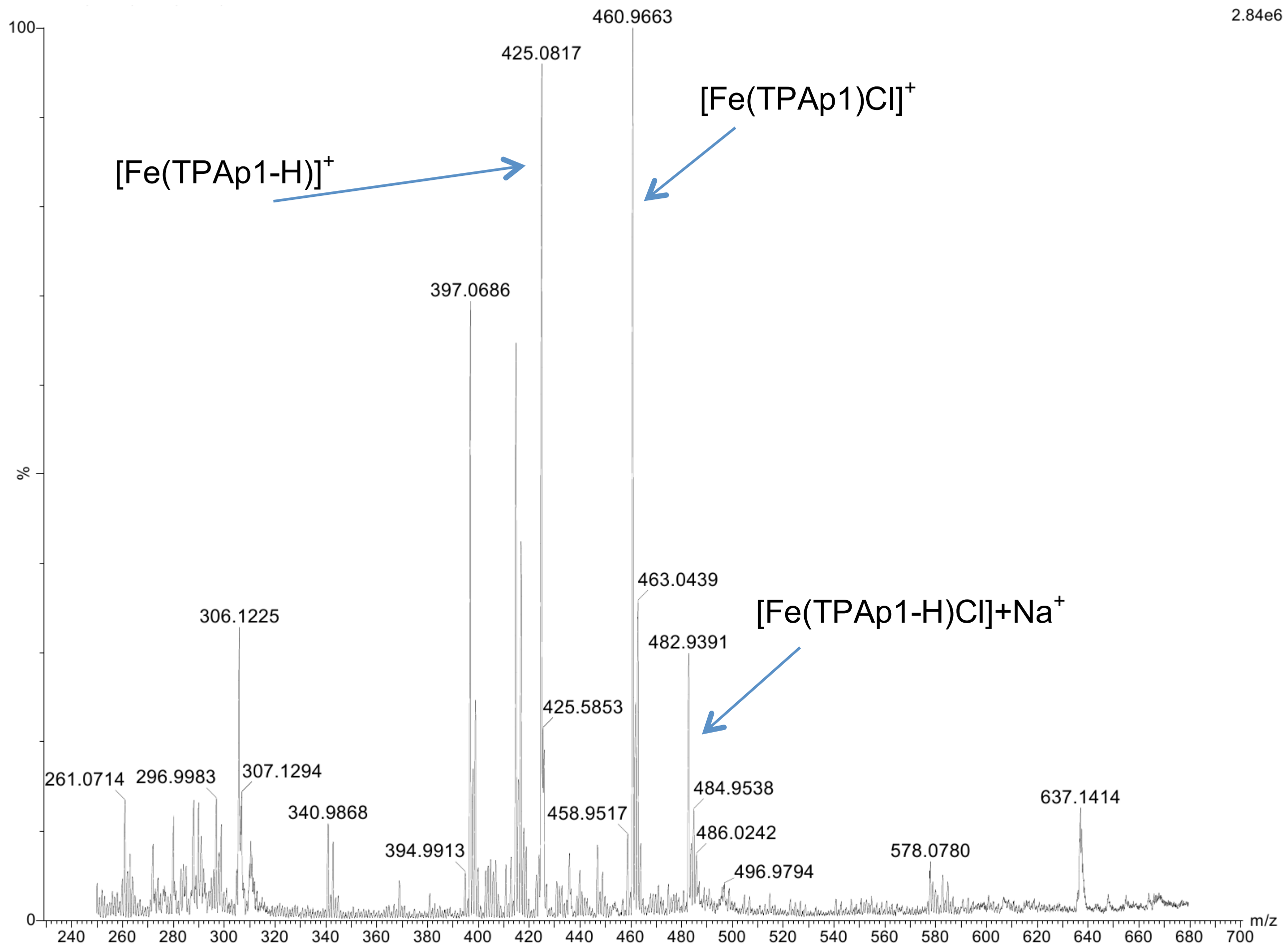

Figure S8. ESI-MS (positive ion mode) of **FeP** in MeOH.

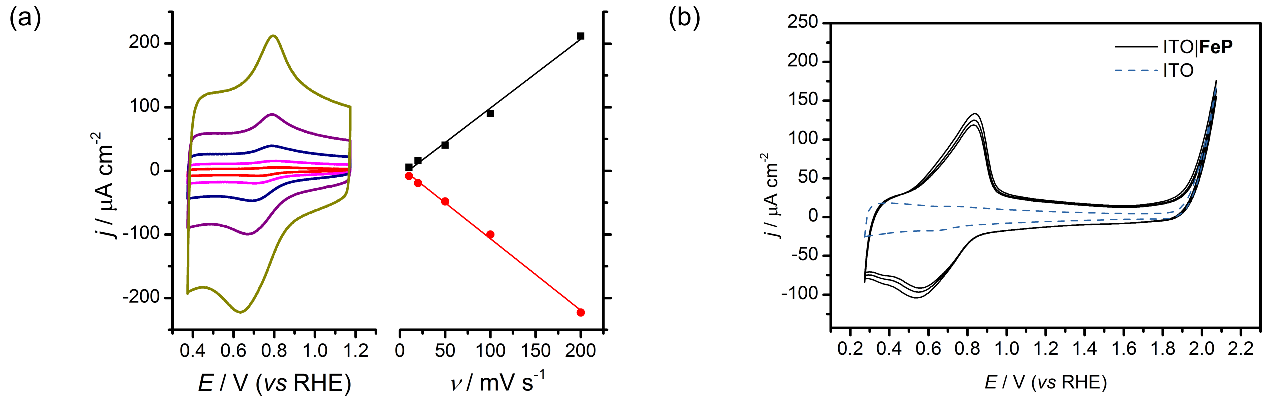

Figure S9. (a) CV of **FeP** on mesoITO at scan rates from ν = 10 mV s^{-1} to 200 mV s^{-1} alongside the scan-rate-dependence of the peak current Conditions: aqueous Na_2SO_4 (0.1 M) solution at pH 3 with a Ag/AgCl reference electrode and a Pt mesh counter electrode.

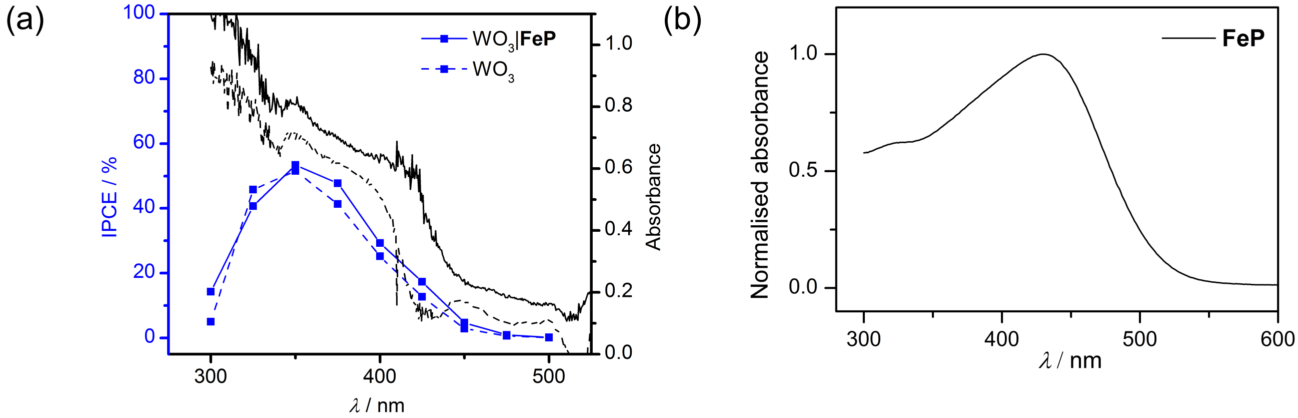

Figure S10 (a) UV/vis spectra (reflectance mode) (black line) and incident photon-to-current efficiency (IPCE, blue lines) of WO_3|**FeP** (solid lines) and WO_3 (dashed lines). Conditions for IPCE: pH 3 Na_2SO_4 (0.1 M), E_{appl} = 1.0 V vs RHE, Pt CE, Ag/AgCl RE, monochromatic irradiation (FWHM = 5 nm). (b) UV/vis spectrum of **FeP** in MeOH.

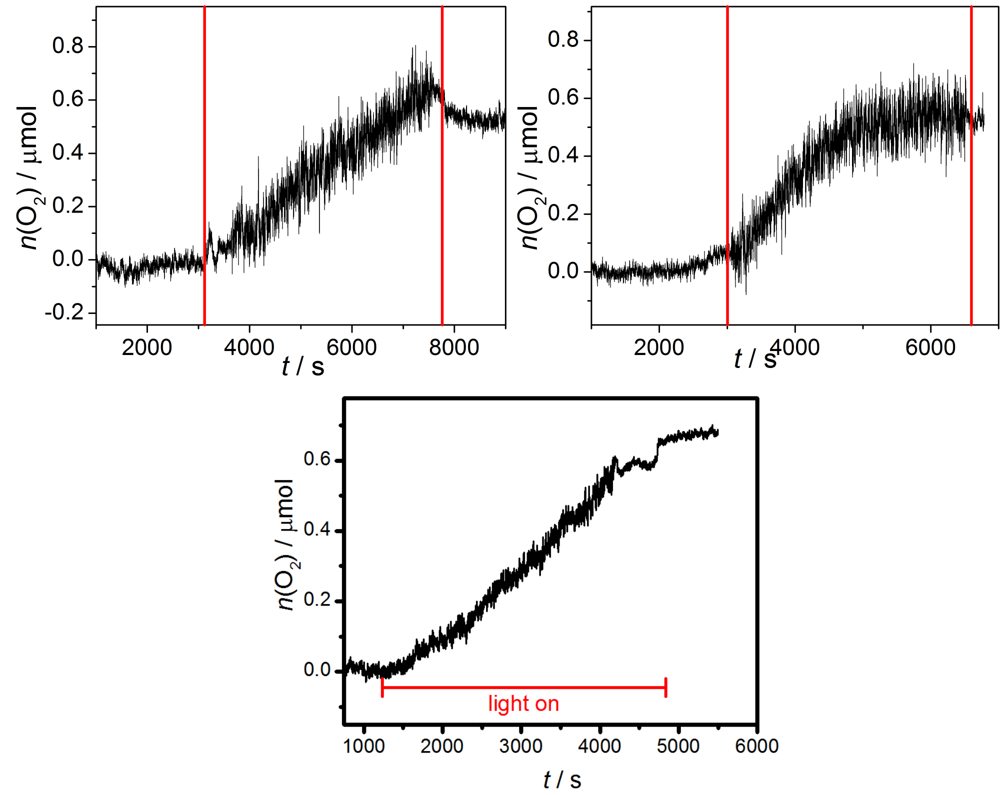

Figure S11. Examples of O_2 detection experiments for the TiO_2|**NiP** / WO_3|**FeP** PEC cell with an applied bias (U_{appl}) of 1.1 V for 1 h under solar illumination. Vertical lines indicate light on and off.

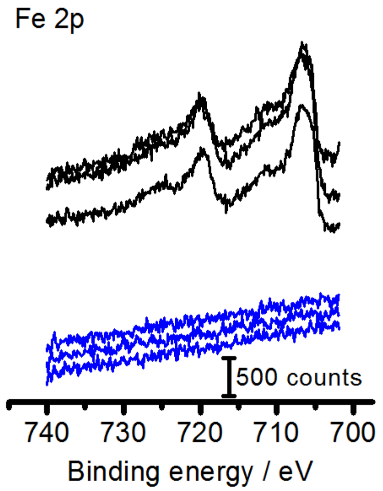

Figure S12. XPS spectrum in the Fe 2p region of WO_3|**FeP** before (black line) and after (blue line) 1 h PEC electrolysis in combination with TiO_2|**NiP** at U_{appl} = 1.1 V under solar illumination.

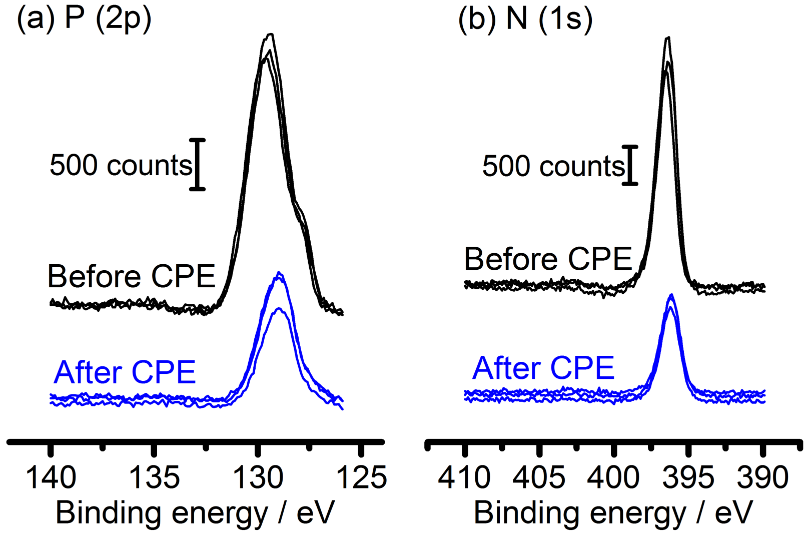

Figure S13 XPS spectrum in the (a) P 2p and (b) N 1s regions of TiO_2|**NiP** before (black line) and after (blue line) 1 h PEC electrolysis in combination with WO_3|**FeP** at U_{appl} = 1.1 V under solar illumination.

Table S1. Relative elemental abundances of WO_3|**FeP** and TiO_2|**NiP** before and after photoelectrolysis for 1 h with an applied bias of 1.1 V derived from XPS data.

	**Before electrolysis**		**After electrolysis**	
	WO_3|**FeP**	TiO_2|**NiP**	WO_3|**FeP**	TiO_2|**NiP**
**Ni**	n/a	1	n/a	1
**Fe**	1	n/a	0	n/a
**P**	1	8.10	0	7.96
**N**	3.82	4.09	0	4.58

Table S2. XPS peak positions (in eV) of WO_3|**FeP** and TiO_2|**NiP** before and after photoelectrolysis for 1 h with an applied bias of 1.1 V.

	**Before electrolysis**		**After electrolysis**	
	WO_3|**FeP**	TiO_2|**NiP**	WO_3|**FeP**	TiO_2|**NiP**
**Ni**	n/a	851.5, 868.9	n/a	851.5,869.1
**Fe**	706.5, 720	n/a	-	n/a
**P**	129	129.4	-	129.0
**N**	396.3	396.4	-	396.2

End of Electronic Supporting Information
